# Influence of combinations of fenugreek, garlic, and black pepper powder on production traits of the broilers

**DOI:** 10.14202/vetworld.2016.470-474

**Published:** 2016-05-11

**Authors:** A. Kirubakaran, M. Moorthy, R. Chitra, G. Prabakar

**Affiliations:** 1 Veterinary University Training and Research Centre, Erode - 638 004, Tamil Nadu, India; 2 Department of Poultry Science, Veterinary College and Research Institute, Namakkal - 637 002, Tamil Nadu, India; 3 Department of Animal Husbandry Statistics and Computer Applications, Veterinary College and Research Institute, Namakkal - 637 002, Tamil Nadu, India

**Keywords:** black pepper, body weight, feed conversion ratio, fenugreek, garlic

## Abstract

**Aim:**

To study the effects of combinations of fenugreek (*Trigonella foenum-graecum* L.), garlic (*Allium sativum*), and black pepper (*Piper nigrum*) powder supplementation on production traits of broiler chickens.

**Materials and Methods:**

A total of 288 commercial broiler chicks were randomly assigned to 1-9 groups with 4 replicates each. An experiment was conducted in broilers with different feed formulations; control feed, with no added fenugreek, garlic, and black pepper powder; and 8 treatment groups receiving feed supplemented with different combinations of fenugreek, garlic, and black pepper powder. The individual broilers’ body weight and feed consumption were recorded and calculate the body weight gain and feed conversion ratio (FCR).

**Results:**

Broiler’s weight gain and FCR were significantly higher in groups receiving feed supplemented with garlic and black pepper powder combinations (p<0.01). Cumulative feed consumption was significantly higher in groups receiving feed supplemented with garlic and black pepper powder combinations (p<0.01).

**Conclusion:**

The combination of garlic and black pepper powder supplemented broiler feed fed groups showed higher production performance. The 5 g/kg garlic powder+1 g/kg black pepper powder and 10 g/kg garlic powder+2 g/kg black pepper powder significantly improved the weight gain and FCR.

## Introduction

Antibiotics are used to control the disease and infection effect in the poultry industry. The commercial broilers are genetic engineering birds, and it can attain maximum body weight (above 2 kg) during its short growing time interval of 35 days. The commercial broilers faced much stress during its growing period, due to attain maximum body weight in shorter duration interval. The poultry nutritionists are added stress relieving medicines to feed to trying to ease stress from broilers and improve the body weight. Large number of laboratory derived antistress and growth promoting medicines are available in the field to improve its performance. However, the use of chemical products, especially hormones and antibiotics, may cause adverse side effects. Attempts to use the natural resources such as medicinal plants could be widely accepted as feed additives to improve the efficiency of feed utilization and productive performance in poultry.

Antibiotic have a certain period as a pulling out time. If the antibiotics are not reserved from the broiler diet before catching the birds for slaughter, it will lead to a problem, like deposition of antibiotic residues in commercial broiler meats and also delivered the antibiotic residues to the consumers through this meat consumption. Poultry nutritionists are trying to rectify this problem through supplementation of phytoadditives, which contain antibiotic and antibacterial properties. The majority of medicinal plants do not have the residual effects [1].

Fenugreek, garlic, and black pepper plants contain beneficial phytoadditives in its parts. Their phytochemical efficiency was also analyzed by many researchers, and there was a great need to judge its efficacy and economic feasibility on the basis of different dosage levels in the broiler diet. The broilers’ body weight was significantly (p<0.01) increased in fenugreek seed powder (0, 10, 20, and 40 g/kg) fed groups compared to control (1382, 2587, 2328, and 2192.5 g/bird) and observed significantly (p<0.01) decreased feed consumption and significant (p<0.01) improvement in feed conversion ratio between the groups [2]. The some researchers were reported the addition of fenugreek powder and extract to diet has improved the body weight gain in broilers [3,4]. The 42 days trial by feeding garlic powder at the rate of 0, 2 and, 4 g/kg in commercial broiler diet and found no significant effects on body weight and weight gain, no difference in feed consumption and feed efficiency during the experimental period [5]. The addition of garlic to the diet has improved the production performance of broiler [6-8]. The black pepper powder was added at the rate of 0, 5, 7.5, and 10 g/kg in broiler diet and noted significant (p<0.05) difference in body weight (1855, 1990, 2025, and 2144 g/bird), body weight gain (1810, 1945, 1980, and 2099 g/bird), increased feed consumption (3620, 3793, 3841, and 4030 g/bird), and positive significant (p<0.05) effect on feed conversion ratio (2.00, 1.95, 1.94, and 1.92) at 42 days of age [9]. Some researchers were found no difference in feed intake in broiler fed with black pepper for a period of 5-week [10,11].

The individual phytoadditives are used as an antibiotic replacer in poultry field. In these circumstances, combinations of fenugreek (*Trigonella foenum-graecum* L.), garlic (*Allium sativum*), and black pepper (*Piper nigrum*) are added to broiler in these trials and evaluated its efficacy.

## Materials and Methods

### Ethical approval

The biological trial was carried out at the Poultry Farm Complex and the Department of Poultry Science, Veterinary College and Research Institute (Namakkal, India) after permission of Institutional Animal Ethics Committee.

### Experimental design and dietary treatments:

A total of 288 commercial broiler chicks at day old age, belonging to the same hatch and of uniform body size, were randomly assigned to 9 dietary treatments. Four replicates were randomly assigned to each of the 9 dietary treatments. Eight chicks were allotted to each of the replicates. The broilers are vaccinated with live B1 and Lasota strain on the end of 1^st^ week and end of 3^rd^ week. The experimental designs are showed in Table-1.

**Table-1:** Experimental design.

Treatment groups	Particulars	Number of replicates	Number of birds/replicate	Total
T_1_	Control−standard broiler diet	4	8	32
T_2_	Control+5 g fenugreek powder/kg of diet+5 g garlic powder/kg of diet	4	8	32
T_3_	Control+10 g fenugreek powder/kg of diet+10 g garlic powder/kg of diet	4	8	32
T_4_	Control+5 g fenugreek powder/kg of diet+ 1 g black pepper powder/kg of diet	4	8	32
T_5_	Control+10 g fenugreek powder/kg of diet+ 2 g black pepper powder/kg of diet	4	8	32
T_6_	Control+5 g garlic powder/kg of diet+1 g black pepper powder/kg of diet	4	8	32
T_7_	Control+10 g garlic powder/kg of diet+2 g black pepper powder/kg of diet	4	8	32
T_8_	Control+5 g fenugreek powder/kg of diet+5 g garlic powder/kg of diet+ 1 g black pepper powder/kg of diet	4	8	32
T_9_	Control+10 g fenugreek powder/kg of diet+10 g garlic powder/kg of diet+2 g black pepper powder/kg of diet	4	8	32
Total				288

Samples of fenugreek, garlic, and black pepper used in the experimental feeds were assayed in duplicate [12]. The experimental diet was formulated according to the standards prescribed in Bureau of Indian Standards [13]. The locally available fenugreek, garlic, and black pepper were purchased and powdered and incorporated into standard broiler diet to form different experimental diets. The phytoaddditives, fenugreek, garlic, and black pepper combinations are added as feed supplements and act as antibiotic alternatives.

The total phenolic content of fenugreek powder was estimated and expressed as mg/l gallic acid equivalent of phenols [14]. Total flavonoid content of fenugreek powder was determined and expressed as mg/l quercetin equivalents of flavonoid [15]. The tannin content of fenugreek was determined and calculated as mg/l tannic acid equivalents of tannin [16]. Total phenolic content of garlic powder was determined in the garlic using the Folin-Ciocalteu method [14]. The total flavonoid content of garlic powder was determined expressed as micrograms of rutin equivalents per gram dry weight [17]. Total phenols and flavonoids contents in the black pepper powder extracts were also analyzed by UV spectroscopy [18,19].

### Sample collection and analysis

#### Production traits

The body weight of the individual experimental bird was recorded on initial and weekly to 1-g accuracy from 1 to 6 weeks of age to determine the body weight gain. Replicate-wise feed intake and mortality (if any) were recorded to calculate the feed efficiency.

### Statistical analysis

Data on the 9 dietary treatments were analyzed statistically by one-way ANOVA to determine whether a significant difference existed between the 9 different diets. Significance was tested by a *post-hoc* analysis method.

## Results

The proximate composition (% dry matter basis) and phytochemical concentration of fenugreek, garlic, and black pepper are presented in Table-2.

**Table-2:** Proximate composition (% DM basis) of fenugreek, garlic, and black pepper.

Nutrients	Fenugreek	Garlic	Black pepper
Moisture	7.18	6.10	11.23
Crude protein	28.58	15.93	11.55
Ether extract	7.55	1.30	7.93
Crude fiber	6.27	10.12	12.32
Total ash	2.39	7.31	3.93
Gross energy (kcal/kg)	4500	3797	4015
Metabolizable energy* (kcal/kg)	3877	1490	2550
Phytochemical concentration			
Phenol	110 mg/l	42 mg GAE/100 g	1.728 mg/g
Flavonoid	410 mg/l	0.39 mg rutin DW/g FW	1.087 mg/g
Tannin	100 mg/l	-	-

* Calculated values. DM=Dry matter, DW=Dry weight, GAE=Gallic acid equivalent

### Production traits

Comparisons for production traits, body weight, body weight gain, feed consumption, and feed conversion ratio are shown in Tables-3-6.

**Table-3:** Mean±SE body weight (g) of broilers fed with different combinations of fenugreek, garlic, and black pepper.

Weeks	T_1_	T_2_	T_3_	T_4_	T_5_	T_6_	T_7_	T_8_	T_9_	p value
Hatch weight	48.56±0.63	48.02±0.57	47.02±0.54	48.94±0.67	47.75±0.60	48.06±0.65	48.10±0.47	48.07±0.60	48.12±0.67	0.6562
1^st^ week	118.56^D^±2.64	120.06^D^±1.98	119.00^D^±2.04	125.09^CD^±2.71	121.09^D^±1.43	144.03^A^±1.81	149.40^A^±1.68	130.00^BC^±1.78	135.00^B^±1.39	<0.0001
2^nd^ week	282.96^F^±6.49	300.90^DEF^±5.18	290.59^EF^±4.89	310.84^CDE^±7.04	305.15^DE^±3.85	350.00^AB^±3.27	360.00^A^±5.22	320.03^CD^±3.56	330.12^BC^±2.90	<0.0001
3^rd^ week	560.75^C^±13.37	575.06^BC^±10.47	570.65^BC^±10.30	618.03^ABC^±11.30	603.18^ABC^±10.39	650.03^A^±13.65	660.00^A^±12.78	625.50^AB^±19.30	635.28^A^±17.66	0.0001
4^th^ week	880.81^D^±21.75	900.90^BCD^±13.85	890.00^CD^±19.51	931.78^ABCD^±17.54	924.15^ABCD^±14.13	972.90^A^±12.26	980.00^A^±7.27	950.71^ABC^±13.82	967.25^AB^±14.98	0.0001
5^th^ week	1295.93^C^±16.90	1300.00^C^±39.83	1295.18^C^±23.07	1410.15^ABC^±37.70	1369.84^BC^±20.90	1450.62^AB^±30.83	1490.62^A^±11.54	1425.46^AB^±21.33	1437.65^AB^±19.36	<0.0001
6^th^ week	1785.34^D^±40.2	1801.37^D^±28.88	1798.84^D^±41.41	1905.09^CD^±28.08	1896.84^CD^±26.77	2053.12^AB^±14.88	2090.62^A^±17.01	1915.28^CD^±33.91	1946.25^BC^±24.70	<0.0001

Values given in each cell is the mean of 32 observations.

A-F Means within a row bearing different superscripts differ significantly (p<0.01). SE=Standard error

**Table-4:** Mean±SE body weight gain (g) of broilers fed with different combinations of fenugreek, garlic, and black pepper.

Weeks	T_1_	T_2_	T_3_	T_4_	T_5_	T_6_	T_7_	T_8_	T_9_	p value
1^st^ week	69.99^E^±2.70	72.04^E^±2.09	71.97^E^±1.93	76.15^DE^±2.88	73.34^DE^±1.66	95.96^AB^±1.91	101.30^A^±1.63	81.92^CD^±1.98	86.87^BC^±1.53	<0.0001
2^nd^ week	234.40^F^±6.38	252.88^DEF^±5.15	242.90^EF^±4.97	261.90^CDE^±7.31	257.40^DE^±3.94	301.93^AB^±3.27	311.90^A^±5.28	271.95^CD^±3.84	281.99^BC^±2.99	<0.0001
3^rd^ week	512.18^D^±13.38	527.04^BCD^±10.48	522.97^CD^±10.30	569.08^ABCD^±11.41	555.43^ABCD^±10.46	601.96^A^±13.70	611.90^A^±12.79	577.42^ABC^±19.41	587.15^AB^±17.73	0.0001
4^th^ week	832.24^D^±21.80	852.88^BCD^±13.97	842.31^CD^±19.44	882.83^ABCD^±17.71	876.96^ABCD^±14.18	924.84^A^±12.32	931.90^A^±7.40	902.64^ABC^±13.92	919.12^AB^±14.93	0.0005
5^th^ week	1247.37^C^±16.91	1251.97^C^±39.76	1247.50^CD^±23.00	1361.21^ABC^±37.89	1322.65^BC^±21.18	1402.55^AB^±30.76	1442.52^A^±11.42	1377.39^AB^±21.48	1389.52^AB^±19.13	<0.0001
6^th^ week	1736.27^D^±40.14	1753.35^D^±28.97	1751.15^D^±41.40	1856.15^CD^±28.37	1849.65^CD^±26.96	2005.05^AB^±15.03	2042.52^A^±17.09	1867.20^CD^±34.01	1898.12^BC^±24.52	<0.0001

Value given in each cell is the mean of 32 observations.

A-F Means within a row bearing different superscripts differ significantly (p<0.01). SE=Standard error

**Table-5:** Mean±SE cumulative feed consumption (g) of broilers fed with different combinations of fenugreek, garlic, and black pepper.

Weeks	T_1_	T_2_	T_3_	T_4_	T_5_	T_6_	T_7_	T_8_	T_9_	p value
1^st^ week	93.21^C^±2.30	79.50^E^±0.08	78.00^E^±0.76	84.75^D^±0.34	79.46^E^±0.92	101.96^B^±0.33	110.00^A^±1.68	91.50^E^±0.68	95.81^E^±0.89	<0.0001
2^nd^ week	336.96^BC^±2.77	294.50^FG^±8.74	280.00^G^±2.20	303.75^EF^±0.91	298.21^F^±3.98	349.71^AB^±0.87	360.37^A^±5.07	319.06^DE^±2.41	328.31^CD^±0.60	<0.0001
3^rd^ week	770.40^A^±9.74	672.00^E^±8.55	660.00^E^±6.67	724.96^CD^±6.76	706.81^D^±4.35	765.21^AB^±1.18	768.50^A^±5.91	740.31^BC^±1.90	748.00^ABC^±5.79	<0.0001
4^th^ week	1329.14^A^±14.93	1176.71^DE^±12.08	1160.00^E^±9.05	1215.96^CD^±7.95	1211.18^CD^±6.64	1275.84^B^±4.71	1275.81^B^±3.82	1250.31^BC^±1.90	1270.90^B^±11.95	<0.0001
5^th^ week	2226.33^A^±14.62	2040.78^C^±10.54	2039.06^C^±9.98	2070.34^C^±12.43	2065.56^C^±13.43	2091.78^BC^±9.86	2136.37^B^±7.65	2090.37^BC^±7.63	2086.21^BC^±18.70	<0.0001
6^th^ week	3076.33^B^±22.16	2960.15^D^±12.64	2950.00^D^±13.20	2979.71^CD^±18.27	2970.09^CD^±13.11	3185.53^A^±9.04	3236.37^A^±7.65	2989.43^CD^±8.28	3024.18^BC^±18.57	<0.0001

Values given in each cell is the mean of four observations.

A-F Means within a row bearing different superscripts differ significantly (p<0.01). SE=Standard error

**Table-6:** Mean±SE cumulative feed conversion ratio of broilers fed with different combinations of fenugreek, garlic, and black pepper.

Weeks	T_1_	T_2_	T_3_	T_4_	T_5_	T_6_	T_7_	T_8_	T_9_	p value
1^st^ week	1.35^B^±0.05	1.13^A^±0.04	1.11^A^±0.03	1.16^A^±0.04	1.10^A^±0.03	1.07^A^±0.02	1.09^A^±0.03	1.13^A^±0.02	1.11^A^±0.02	0.0003
2^nd^ week	1.44^B^±0.05	1.18^A^±0.04	1.16^A^±0.02	1.19^A^±0.04	1.16^A^±0.02	1.16^A^±0.01	1.16^A^±0.02	1.18^A^±0.01	1.16^A^±0.01	<0.0001
3^rd^ week	1.53^B^±0.04	1.29^A^±0.03	1.28^A^±0.03	1.29^A^±0.03	1.28^A^±0.03	1.29^A^±0.03	1.27^A^±0.03	1.30^A^±0.05	1.31^A^±0.04	0.0037
4^th^ week	1.60^B^±0.04	1.39^A^±0.03	1.40^A^±0.04	1.39^A^±0.04	1.39^A^±0.03	1.38^A^±0.03	1.30^A^±0.01	1.39^A^±0.02	1.39^A^±0.02	0.0002
5^th^ week	1.79^D^±0.03	1.68^CD^±0.05	1.65^BCD^±0.03	1.56^ABC^±0.04	1.57^ABC^±0.03	1.51^AB^±0.03	1.48^A^±0.01	1.53^ABC^±0.02	1.51^AB^±0.02	<0.0001
6^th^ week	1.80^B^±0.04	1.70^AB^±0.03	1.71^AB^±0.04	1.61^A^±0.03	1.62^A^±0.03	1.59^A^±0.01	1.58^A^±0.01	1.61^A^±0.03	1.60^A^±0.02	0.0010

Value given in each cell is the mean of four observations.

A-D Means within a row bearing different superscripts differ significantly (p<0.01). SE=Standard error

## Discussion

### Body weight and weight gain

The analysis of variance of data on mean body weight (g) and body weight gain of broilers revealed significant (p<0.01) difference from 1 to 6 weeks of age between treatment groups due to dietary supplementation of fenugreek, garlic, and black pepper combinations (Tables-3 and 4). Fenugreek and garlic combinations (T_2_ and T_3_) revealed numerically higher body weight and weight gain than the control group at 6 weeks of age. This might be due to the presence of phytochemical, namely, allicin in garlic powder which improved digestibility and eradicates the pathogenic microbes in the intestine [20]. Fenugreek and black pepper combination fed group (T_4_ and T_5_) showed numerically higher body weight when compared to control group at 6 weeks of age. The higher body weight might be due to the active principle-piperine present in black pepper which has a digestive stimulatory effect. The higher body weight was observed in T_4_ than T_5_. The high proportion (40%) of soluble fiber in the fenugreek seed which forms a gelatinous structure, which might slow down the digestion and absorption of feed from the intestine and create a sense of fullness in the abdomen and thus suppressed appetite and resulted in more weight loss in T_5_ than T_4_ [21].

In garlic and black pepper combination (T_6_ and T_7_), fed broilers showed a significantly (p<0.01) higher body weight when compared to control. This might be due to the synergistic action of garlic phytochemical – allicin and black pepper phytochemical – piperine, which give better results in T_6_ and T_7_. Some researchers were observed significantly higher body weight and weight gain in broilers fed with individual black pepper only at 6 weeks of age [22-28]. The addition of garlic to broiler diet had increased the salivary flow rate and gastric juice secretion which resulted in improved digestibility and higher body weight [29].

The fenugreek, garlic, and black pepper combination group (T_8_ and T_9_) revealed lower body weight than T_6_ and T_7_. This might be due to the presence of fenugreek powder in diet T_8_ and T_9_.

### Feed consumption

The statistical analysis revealed significant (p<0.01) difference between treatment groups in mean feed consumption and feed conversion ratio (Tables-5 and 6) at the end of every week due to dietary supplementation of fenugreek, garlic, and black pepper combinations. The mean cumulative feed consumption was significantly (p<0.01) higher in T_6_ and T_7_ group (3185.53 and 3236.37 g) when compared to control at 6 weeks of age. Similarly, significantly (p<0.01) superior feed conversion ratio was observed in T_6_ and T_7_ (1.59 and 1.58) when compared to control at the end of the experiment. This might be due to the positive synergism between active principles of garlic and black pepper, which resulted in better feed conversion ratio in T_6_ and T_7_ at 6 weeks of age.

## Conclusion

This study clarified that the birds fed rations supplemented with garlic and black pepper combinations utilized their feed more efficiently resulting in higher body weight and also combinations of 5 g garlic+1 g black pepper and 10 g garlic+2 g black pepper powder had resulted in better feed consumption and feed conversion ratio in broilers.

## Authors’ Contributions

This study is the part of Ph.D. thesis of the first author AK, who carried out the research under the guidance of Professor MM. RC and GP helped during the trial. The article was drafted by AK. The revision was made by MM, RC and GP.

All authors have read and approved the final version of the manuscript.
